# A homozygous variant in the *GJB2* gene as a potential phenocopy of Waardenburg syndrome: case report

**DOI:** 10.3389/fmed.2026.1925409

**Published:** 2026-10-07

**Authors:** Yuehua Wu, Yupeng Chen, Zimeng Guo, Lang Xie, Tao Yang, Longnian Li

**Affiliations:** 1 Department of Dermatology, First Affiliated Hospital of Gannan Medical University, Joint Organization of Jiangxi Clinical Medicine Research Center for Dermatology, Ganzhou, Jiangxi, China; 2 The First Clinical Medical College, Gannan Medical University, Ganzhou, Jiangxi, China

**Keywords:** deafness autosomal recessive, *GJB2* gene, homozygous variant, phenocopy, waardenburg syndrome

## Abstract

Waardenburg syndrome (WS) is a neurocristopathy primarily characterized by congenital hearing loss and multisystem pigmentary abnormalities. We report a 12-year-old Han Chinese girl who presented with moderate-to-severe sensorineural hearing loss, a wide nasal root, diffuse hypopigmentation of the hair, and facial freckles—features partially similar to the WS phenotype. However, her calculated W-index was 1.87, below the diagnostic threshold for dystopia canthorum (>1.95), and her overall clinical presentation did not meet the diagnostic criteria for any WS subtype. Whole-exome sequencing (WES) and subsequent familial Sanger sequencing identified a homozygous *GJB2* variant, c.109G > A (p.Val37Ile), in the proband, with both parents confirmed as heterozygous carriers. Based on the integrated clinical and genetic evidence, the patient was diagnosed with *GJB2*-related autosomal recessive nonsyndromic hearing loss (DFNB1A). Her WS-like features were interpreted as a possible phenocopy. This case highlights that in patients with hearing loss exhibiting incomplete or atypical WS features, comprehensive genetic testing—including analysis of *GJB2*—is crucial to differentiate phenocopies, establish an accurate etiology, and provide a basis for precise diagnosis and genetic counseling.

## 1 Introduction

Waardenburg syndrome (WS), also known as audio-pigmentary syndrome, is a neurocristopathy classically characterized by congenital sensorineural hearing loss and multisystem pigmentary abnormalities involving the skin, hair, and iris (1). WS is recognized to be clinically and genetically heterogeneous (2). Genetically, pathogenic variants in genes such as *PAX3*, *MITF*, *SOX10*, *EDNRB*, and *EDN3* have been identified as causative for WS (3). Nevertheless, the clinical presentation in a considerable proportion of patients cannot be attributed to variants in these known genes (4), suggesting the potential involvement of other undiscovered disease-causing genes or the existence of atypical mechanisms, including phenocopy.

It is noteworthy that the *GJB2* gene (encoding gap junction beta-2 protein, connexin 26), while it is the most common cause of autosomal recessive nonsyndromic hearing loss (DFNB1A; MIM #220290) (5), is not a causative gene for WS. Connexin 26, the protein encoded by this gene, is highly expressed in cochlear epithelial cells and supporting cells such as fibrocytes, in which it mediates potassium ion recycling in the endolymph to maintain the electrochemical gradient essential for hearing. Dysfunction of this protein thus leads to sensorineural hearing loss (6). It has been reported that homozygous pathogenic variants in *GJB2* may occasionally be accompanied by WS-like features such as heterochromia iridis and dystopia canthorum, constituting a phenocopy of WS (7). Here we report the case of a 12-year-old girl with a phenotype partially similar to WS who was found to carry a homozygous *GJB2* variant, c.109G > A (p.Val37Ile). This report aims to discuss the clinical and genetic significance of recognizing such phenocopies in patients presenting with congenital hearing loss accompanied by pigmentary abnormalities.

## 2 Case report

A 12-year-old Southern Han Chinese girl was referred for evaluation of hearing loss, noted since infancy, with her family reporting worsening over the preceding six months. Physical examination revealed fine, sparse hair with a diffuse reddish-brown discoloration, a broad nasal root, and symmetrically distributed, scattered brown freckles on the nose and bilateral cheeks (Figures 1A,B). Her clinical presentation suggested the need for differential diagnosis of WS and related deafness-dermatological syndromes. To evaluate whether the broad nasal root constituted dystopia canthorum, her W-index was calculated to be 1.87, which did not meet the diagnostic threshold for dystopia canthorum (>1.95). Audiological assessment confirmed bilateral moderate-to-severe sensorineural hearing loss on pure-tone audiometry. Tympanometry showed type “C” tympanograms bilaterally with absent ipsilateral acoustic reflexes. Hematological parameters and color Doppler ultrasonographic examinations of the heart and abdomen revealed no significant abnormalities. The patient denied any long-term medication history or specific environmental exposures, and was well-nourished. Evaluation of the proband's parents and younger brother identified no hearing impairment or physical stigmata associated with WS, such as broad nasal root or facial freckles; all had black hair. Additionally, the family reported that the patient had academic challenges, potentially related to her hearing impairment.

**Figure 1:**
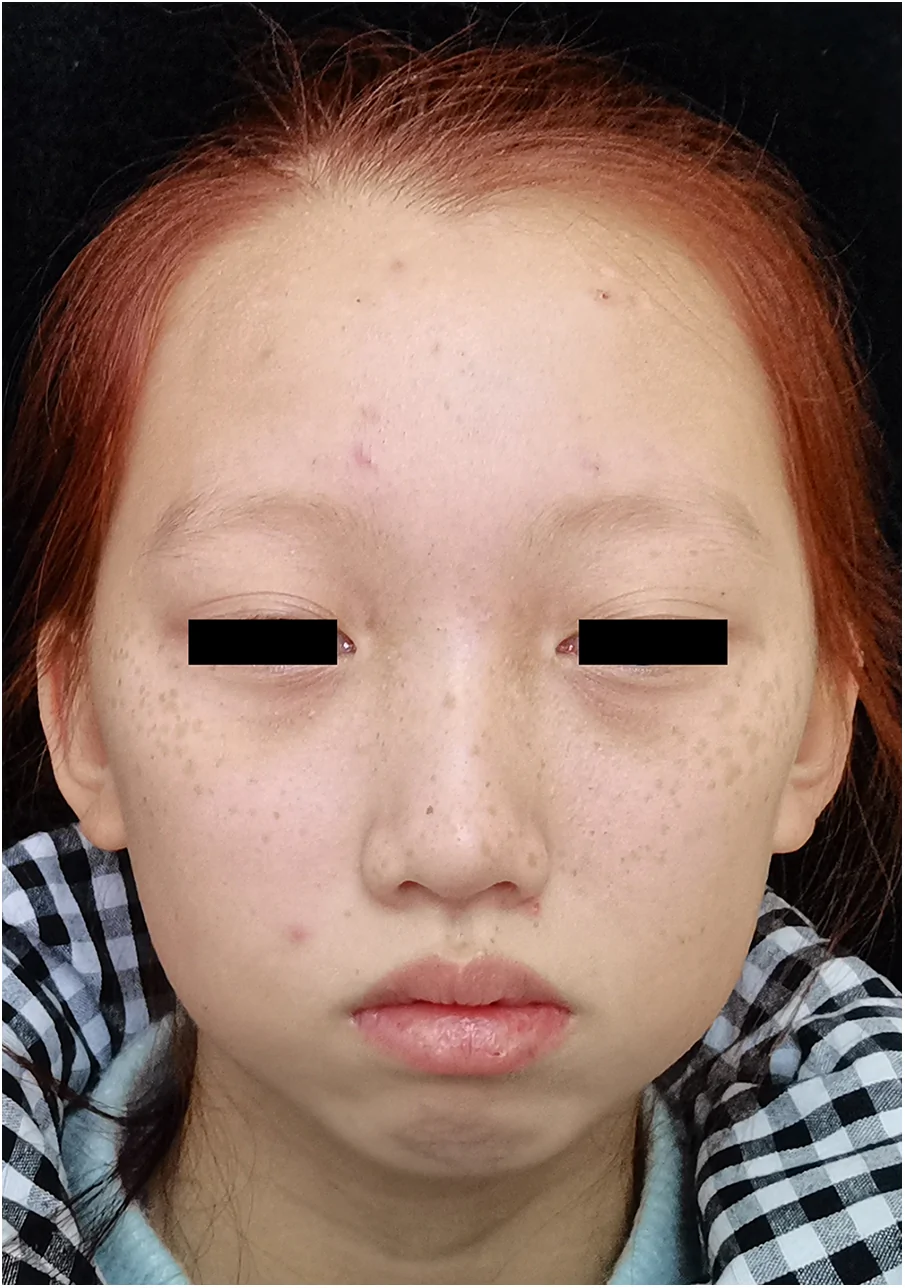
The proband's diffuse reddish-brown hair, broad nasal root, and facial freckling.

After obtaining informed consent, peripheral blood samples were collected from the proband and her family members for genomic DNA extraction. Whole-exome sequencing of the proband detected a pathogenic variant in the *GJB2* gene, c.109G > A (p.Val37Ile). Subsequent Sanger sequencing confirmed the variant and determined its segregation within the family. The results demonstrated that the proband was homozygous for the variant, both parents were heterozygous carriers, and her younger brother had the wild-type genotype (Figure 2). The segregation pattern of this variant within the pedigree was consistent with autosomal recessive inheritance. Given the partial phenotypic similarities with WS, sequencing analysis of WS-associated genes (*PAX3*, *MITF*, *SOX10*, *EDNRB*, *EDN3*, *SNAI2*) revealed no pathogenic variants. Analysis of other known deafness genes identified a single heterozygous pathogenic variant in *MPZL2*, a single heterozygous pathogenic variant in *TMC1*, and two independent heterozygous VUSs (variants of uncertain significance) in trans in *CDH23*. All three genes follow an autosomal recessive inheritance pattern, and a heterozygous status alone does not satisfy the requirement of homozygosity or compound heterozygosity for disease manifestation. Ultimately, the proband was diagnosed with autosomal recessive nonsyndromic hearing loss 1A (DFNB1A) caused by pathogenic variants in the *GJB2* gene, as confirmed by whole-exome sequencing and familial Sanger sequencing.

**Figure 2:**
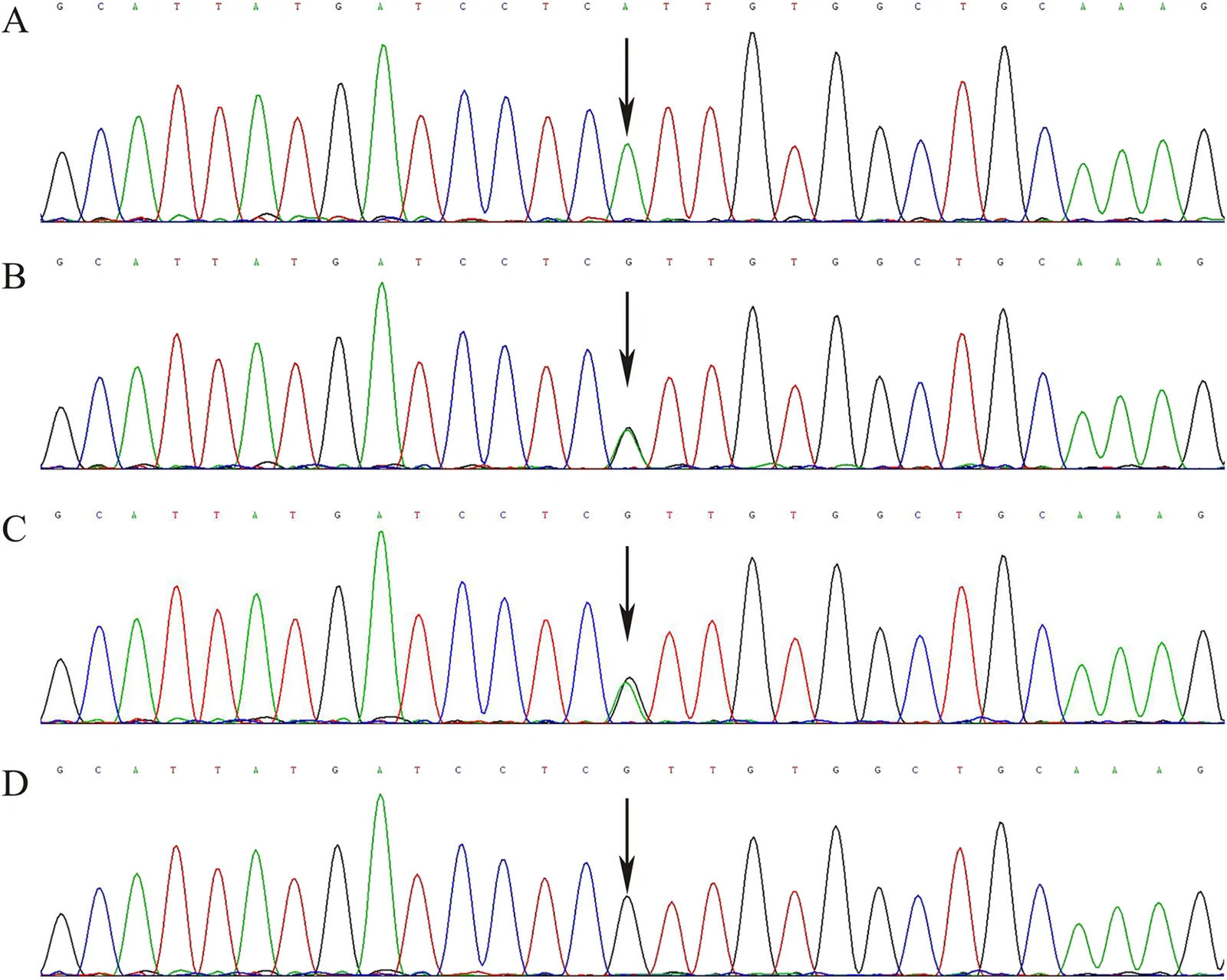
Sanger sequencing validation of the *GJB2* c.109G > A (p.Val37Ile) variant in the family. The arrow indicates the variant site (wild-type: G). **(A)** Proband (homozygous A); **(B)** Father (heterozygous G/A); **(C)** Mother (heterozygous G/A); **(D)** Proband's younger brother (wild-type G).

## 3 Discussion

In terms of hearing phenotype, the ClinGen expert consensus indicates that although *GJB2* p.Val37Ile homozygotes typically present with mild to moderate hearing loss, this variant exhibits variable expressivity and a progressive nature (8). To investigate the phenotypic variability of *GJB2*-related hearing impairment, Chiang et al. identified two genetic mechanisms in *GJB2* p.Val37Ile homozygotes: a minority (approximately 14%) of patients with more severe phenotypes carried pathogenic variants in other hearing loss-related genes; more importantly, a synonymous variant, rs14236, located in the *CRYL1* gene upstream of *GJB2*, is a potential genetic modifier that may lead to worsening hearing loss by increasing the expression level of the *GJB2* p.Val37Ile hypomorphic allele (9). Of note, the *CRYL1* gene itself contains cis-regulatory elements essential for *GJB2* expression (6), providing mechanistic plausibility for its modifying effect. The present patient, a *GJB2* p.Val37Ile homozygote, exhibited moderate-to-severe sensorineural hearing loss, a more severe phenotype than the mild-to-moderate hearing loss typically caused by this variant. Except for the *GJB2* homozygous variant, all variants detected in other hearing loss-related genes were heterozygous and therefore did not meet the homozygous or compound heterozygous criteria for autosomal recessive pathogenicity, and the synonymous variant rs14236 in *CRYL1* was also not detected. Studies have shown that whole-genome sequencing provides a more comprehensive assessment of non-coding region variants compared to whole-exome sequencing (10). Therefore, due to the limitations of whole-exome sequencing, the role of this modifier cannot be completely excluded and remains a possible explanation for the worsening hearing loss in this patient. Furthermore, Chen et al., based on a large-scale screening covering individuals aged 0–97 years, confirmed that hearing loss associated with biallelic *GJB2* p.Val37Ile variants (with no significant difference in hearing thresholds between homozygotes and compound heterozygotes) progressively worsens with age, with the incidence increasing from 9.52% in the 7–15 years age group to 80.00% in the 60–85 years age group (11). The present patient has experienced hearing decline since childhood, which has worsened over the past six months, according to the family. Consistent with the findings of Chen et al., this variant has a clear progressive nature, suggesting that long-term follow-up monitoring of the patient's hearing status may be necessary.

The patient was diagnosed with *GJB2*-related DFNB1A based on whole-exome sequencing and familial Sanger sequencing. However, her clinical presentation—including diffuse reddish-brown hair, a broad nasal root, and facial brown freckles—extends beyond the typical phenotypic spectrum of DFNB1A. Notably, *GJB2* mutations can cause syndromic skin disorders with deafness, including Keratitis-Ichthyosis-Deafness (KID) syndrome, hystrix-like ichthyosis-deafness (HID) syndrome, Vohwinkel syndrome, Bart-Pumphrey syndrome, and palmoplantar keratoderma with deafness, all of which exhibit an autosomal dominant inheritance pattern and are characterized by hyperkeratotic skin lesions (12). In contrast, the present case exhibits an autosomal recessive inheritance pattern, with skin changes limited to scattered brown facial freckles and lacking hyperkeratotic lesions, keratitis, palmoplantar keratoderma, or nail dystrophy. This phenotype is fundamentally distinct from those autosomal dominant disorders characterized by hyperkeratotic manifestations. Therefore, these *GJB2*-related syndromic skin disorders can confidently be excluded. The diffuse reddish-brown hair, representing significant hypopigmentation compared to her parents' black hair, suggests a possible abnormality in hair melanin synthesis. Although not the classic white forelock of WS (one of the major diagnostic criteria), Read et al. noted that the forelock may present as red hair; furthermore, premature graying (defined as predominantly white scalp hair before age 30) is itself a minor diagnostic criterion for WS (1). Therefore, the diffuse reddish hair in this patient does not meet the classic description of either the major or minor diagnostic criteria for WS. Notably, the facial brown freckles do not align with the congenital leukoderma (patchy cutaneous hypopigmentation) described in the Western diagnostic criteria for WS. However, a distinctive phenotypic subtype characterized by cutaneous brown freckles has been reported among Chinese patients with WS2 (13, 14). Given the partial phenotypic similarity to WS, differential diagnosis is warranted. WS1 is characterized by dystopia canthorum (diagnostically defined by a W-index ≥ 1.95), whereas WS2 is defined by its absence (15). WS3 exhibits the phenotypic features of WS1 but is additionally characterized by musculoskeletal abnormalities of the upper limbs (4). WS4 presents with a phenotype similar to WS2 but is associated with Hirschsprung disease (16). Dystopia canthorum was ruled out by a W-index calculation of 1.87 (threshold >1.95), which excludes WS1 and WS3. Furthermore, her constellation of clinical features does not meet the diagnostic criteria for WS2, thereby excluding a diagnosis of WS4; this conclusion is further corroborated by the absence of clinical features suggestive of Hirschsprung disease. Whole-exome sequencing identified no pathogenic variants in known WS-related genes. Therefore, the evidence is insufficient to support a diagnosis of any WS subtype.

Given that the *GJB2* homozygous variant can only explain her sensorineural hearing loss but not the other clinical features (such as diffuse reddish-brown hair, a broad nasal root, and facial brown freckles), the possibility of a phenocopy must be considered. Recent studies suggest that the phenotype of *GJB2*-related hearing loss can be heterogeneous and may occasionally be accompanied by WS-like features, constituting a clinical phenocopy. For instance, Somashekar PH et al. reported a DFNB1A patient with a homozygous *GJB2* c.71G > A [p.(Trp24Ter)] variant, which could explain the hearing loss but not the accompanying clinical features of heterochromia iridis, dystopia canthorum, and synophrys, and this case was ultimately defined as a “phenocopy of WS1′ (7). Unlike the previously reported case, this is the first report of a WS phenocopy associated with the homozygous *GJB2* c.109G > A (p.Val37Ile) variant. The phenotype of our patient (characterized by hair hypopigmentation, facial freckles, and a broad nasal root) differs and does not mimic any specific WS subtype. Nevertheless, both cases represent WS-like phenotypes associated with homozygous *GJB2* variants that extend beyond the DFNB1A presentation, thereby collectively substantiating the existence of this phenocopy phenomenon. This suggests that the phenotypic spectrum of *GJB2*-related disorders may be broader than previously recognized. While homozygous *GJB2* variants can occasionally manifest with diverse WS-like features, the possibility that these features are independent, coincidental traits unrelated to the hearing loss cannot be entirely ruled out. Integrating the clinical and molecular genetic findings, this case is best classified as DFNB1A caused by the homozygous *GJB2* c.109G > A (p.Val37Ile) variant, with the accompanying WS-like features representing a potential phenocopy of WS. This finding has important clinical implications. For patients with hearing loss suspected of having WS, systematic phenotypic evaluation combined with comprehensive genetic testing, including *GJB2* analysis and especially whole-genome sequencing, is crucial for differential diagnosis and etiological determination. In genetic counseling, it is essential to clearly communicate two key points to the family: First, *GJB2*-related DFNB1A is an autosomal recessive disorder, carrying a 25% recurrence risk for future offspring of the carrier parents. Second, this variant has a progressive nature, necessitating long-term follow-up monitoring of hearing function. This case underscores the critical role of integrating systematic phenotyping with comprehensive genetic testing to identify such *GJB2*-related WS phenocopies.

## 4 Conclusion

We report the first case of DFNB1A caused by a homozygous *GJB2* c.109G > A (p.Val37Ile) variant, presenting as a potential phenocopy of WS, further expanding the phenotypic spectrum of *GJB2*-associated disorders. In clinical practice, comprehensive genetic testing including *GJB2* should be performed in patients with hearing loss and pigmentary abnormalities, as it helps distinguish phenocopies, establish a definitive etiological diagnosis, and provide a basis for genetic counseling.

## Data Availability

The datasets presented in this article are not readily available because of ethical/privacy restrictions. Requests to access the datasets should be directed to the corresponding author.
